# Barcoding and mitochondrial phylogenetics of *Porites* corals

**DOI:** 10.1371/journal.pone.0290505

**Published:** 2024-02-15

**Authors:** David J. Combosch, David Burdick, Karim Primov, Dareon Rios, Kireon Rios, Jessica Fernandez

**Affiliations:** Marine Laboratory, University of Guam, Mangilao, Guam; Laboratoire de Biologie du Développement de Villefranche-sur-Mer, FRANCE

## Abstract

Coral reefs are the most diverse ecosystem on the planet based on the abundance and diversity of phyla and higher taxa. However, it is still difficult to assess the diversity of lower taxa, especially at the species level. One tool for improving the identification of lower taxa are genetic markers that can distinguish cryptic species and assess species boundaries. Here, we present one such approach for an important and challenging group of reef-building corals. *Porites* corals are the main reef-builders of many coral reefs in the Indo-Pacific, owing to the massive growth forms of some species. The current number of valid *Porites* species is controversial, inflated with many synonymies, and often based on gross colony morphology although several morphospecies believed to be widespread and common can only be distinguished based on detailed microstructure analyses by taxonomic experts. Here, we test the suitability of multiple regions of mtDNA as genetic barcodes to identify suitable markers for species differentiation and unambiguous identification. Resulting sequencing data was further used for the first phylogenetic analysis of Guam’s *Porites* species. We tested eight different mitochondrial markers and analyzed four in detail for 135 *Porites* specimens: mtDNA markers were amplified for 67 *Porites* specimens from Guam, representing 12 nominal *Porites* species, and combined with 69 mitochondrial genomes, mostly from Hawaii. The combination of all 4 markers distinguished 10 common and 7 uncommon Central-West Pacific *Porites* species. Most clades separate species along taxonomic boundaries, which is uncommon for *Porites* corals and testifies to the suitability of our multi-marker approach, and a combination of the two most promising barcodes distinguished 8/10 common species. These barcodes are thus suitable to distinguish virtually cryptic species in one of the most important and challenging coral genera. They offer a cheap, fast and reliable way to identify *Porites* species for species-level research, monitoring and conservation.

## 1. Introduction

Corals are the main ecosystem engineers of coral reefs, the most diverse marine ecosystem in the world. Millions of people around the world rely on coral reefs for a significant portion of their diet, for coastline protection, and for the cultural significance and economic benefits reefs provide to local economies [1, 2]. Over the last several decades, coral reefs have been declining globally due to a variety of factors, including global climate change, coastal development and deteriorating water quality, and over-fishing [3–7]. This decline has led to a significant increase in coral research, addressing increasingly complex and sophisticated ecologic and evolutionary questions. Virtually all these studies, however, depend on a reliable taxonomic system to identify and classify the species in question. In addition, taxonomy is further essential for any targeted conservation and management approach [8, 9].

Reliable species identification has been a major issue for coral studies, termed “The Species Problem in Corals” [10], and presents a significant obstacle for research, e.g. [11, 12]. Traditionally, coral species identification relies on skeletal morphology [13]. On one hand, however, coral species are often distinguished using overly coarse, whole-colony characteristics, which are convenient for field studies but rarely suitable to reliably identify and distinguish actual species. Significant and widespread phenotypic plasticity has complicated species identification based on macro-morphology even further (e.g. [14]). On the other hand, sophisticated coral species descriptions are available but geographic variation and taxonomic confusion have led to an inflation of described coral species. For example, within this study subject coral genus, *Porites* Link 1807, over 500 species have been described but less than 10% of them are generally considered valid [11, 15] and e.g. the World Register of Marine Species currently lists only 68 valid species [16].

It was largely the application of molecular tools in population and phylogenetic studies that led to the identification and reassessment of actual species boundaries among corals [17]. Phylogenetic analyses revealed numerous discrepancies among morphology-based species descriptions, like cryptic divergences among numerous sympatric congeners (e.g. [18–24]). These discoveries have led to numerous and ongoing taxonomic revisions and/or significant readjustments of morphologic characteristics for species identification (e.g. [25–30].

For some taxa, however, morphologic differences between species are extremely subtle and micro-morphological characters may depend on sophisticated electron microscopy. A simple DNA-based barcoding test is often more convenient and reliable to assess species boundaries and identify cryptic species in many situations. Moreover, molecular phylogenies might reveal previously unrecognized species boundaries, which can then be used to subsequently identify and establish morphological characteristics, e.g. for field studies (e.g. [31]). Therefore, we set out to develop and test simple and straight-forward mitochondrial barcoding markers for the species identification in arguably the most important reef-building genera across the Indo-Pacific, *Porites* corals.

Mitochondrial markers have long been a favorite class for evolutionary geneticists. They tend to be rather straightforward to amplify and sequence due to their predominantly mono-allelic presence in eukaryotes [32–36]. Moreover, mitochondrial DNA (mtDNA) has been found to be highly variable among but also within species in most metazoan phyla [37], which led to numerous phylogenetic and population genetic studies based exclusively on mitochondrial sequence data (e.g. [38–46]).

However, mitochondrial markers are not without challenges. For example, they tend to be less variable in certain taxa, such as in many Anthozoan taxa, including many reef coral genera, which has been hypothesized to be due to purifying selection and/or enhanced mitochondrial DNA repair mechanisms [47–49]. Moreover, mitochondrial markers can give a clean but lopsided impression of the evolutionary history of specimens and taxa due to their matri-lineal inheritance. For example, a single introgressive hybridization event can lead to the sustained presence of distinct mitochondrial lineages within a species. Studies based exclusively on mitochondrial markers would then give the impression of an entirely different species even though generations of “inbreeding” have long purged most of the introgressed DNA in the nuclear genome. There is some evidence for mito-nuclear discordance in *Porites* corals [e.g. 24, 50] and this has recently been found to be widespread among coral in general [51] (see discussion as well).

Members of the *Porites* genus are major structural components in coral reefs [52] due to the massive skeleton structure some species build. *Porites* corals are widespread across most reef environments and frequently dominate brackish and murky habitats where few other corals thrive [53, 54]. *Porites* are also notable for their bleaching resistance, particularly when compared to other dominant coral genera like *Acropora* and *Pocillopora* [55–57]. However, *Porites* corals are notorious for the complexity of their species boundaries and are a prime example of “the species problem” [58]. Species can be massive, branching, encrusting, plating, columnar, and form micro atolls and corallites and several species have been found to grow in multiple colony shapes (e.g. [24, 54, 59, 60]. Moreover, differences in the arrangement and structure of corallites are traditionally used to distinguish between massive species in *Porites* are particularly irregular and highly varied [60, 61], making it difficult or impossible to differentiate them in the field and difficult even to taxonomic experts in the laboratory (pers. observ. and comm.).

Recently genome-wide sequencing markers have been employed to decipher the species boundaries and phylogenetic relationships among *Porites* corals in Hawaii [61] and the Red Sea [50]. However, the taxonomic scope and narrow geographic focus of these studies only provided significant taxonomic clarification for the Red Sea and around the Arabian Peninsula. Pacific *Porites* species continue to remain very challenging to distinguish, despite its enormous ecological role and resulting interest.

Due to these challenges, no unified comprehensive phylogenetic revision of this genus has yet been conducted. However, several studies have generated significant molecular data and we have combined a large amount of published data to identify suitable barcode markers. Moreover, we generated sequence data for eight mitochondrial markers for 67 *Porites* samples from Guam that were identified as 13 different morphospecies (S1 Table). We analyzed four of these markers in detail with a significantly expanded dataset, including 69 published mitochondrial genomes for *Porites* species and seven outgroup taxa, and suggest two mitochondrial markers for barcoding and *Porites* species identification in future studies.

## 2. Methods

### 2.1 Species, samples, sequences

In total, we analyzed 136 *Porites* samples, representing 18 nominal morphospecies and 4 undescribed species (S1 Table). An additional seven outgroup taxa were included for phylogenetic analyses. Only samples with high-quality sequence data for at least two out of the four analyzed markers were included. All analyzed samples are listed in S1 Table, including species name, collection and voucher identifiers, sampling locations, and GenBank accession numbers.

The final dataset consisted of 143 samples from three different sources: 1) 67 *Porites* samples, representing 12 nominal species and 1 undescribed species, were collected on Guam and processed and sequenced as described below. 2) 58 recently published *Porites* mitochondrial genomes, representing 5 nominal species and 3 undescribed species, were obtained from [61]). 3) 18 mitochondrial genomes were obtained from NCBI, including 11 *Porites* mtDNA genomes, representing 9 nominal species, and the following seven outgroup taxa: the poritiid *Goniopora columna* and the dendrophylliids *Turbinaria peltata*, *Dendrophyllia arbuscula*, *D*. *cribrosa*, *Tubastrea tagusensis* as well as two mitochondrial genomes of *T*. *coccinea* (downloaded in December 2019).

Samples from Guam were collected by the authors under a collection permit issued to the University of Guam Marine Lab. Small coral nubbins were collected using SCUBA or free diving and transported alive to the UOG Marine Lab. There, small pieces were preserved in 95% Ethanol and stored at -20°C until further processing. Additional sample material was bleached and preserved as skeleton voucher specimens. Tissue and voucher specimens as well as underwater photographs are available for most samples from Guam in the UOG Marine Lab Biorepository (https://specifyportal.uog.edu/) as indicated in S1 Table.

### 2.2 Molecular methods

Genomic DNA was extracted using GenCatch Genomic DNA Extraction Kits from Epoch Life Science (Sugar Land, TX, USA). Mitochondrial markers were PCR-amplified using primers listed in S2 Table. PCR reactions (25 μl) contained 1 ul of template DNA, 0.75 μl of each primer (10 mM), 0.75 μl of dNTP’s (10 mM; Kapa Biosystems, Wilmington, MA, USA), 0.5 μl of HIFI DNA Polymerase (Kapa Biosystems), 5 μl of 5x PCR buffer (Kapa Biosystems) and molecular grade DI water up to 25 μl. PCR conditions consisted of an initial denaturation step at 94°C for 2min, followed by 30 cycles of 30s at 94°C, 30s at 56–63°C, and 60s at 72°C, and a final 5 min extension step at 72°C. PCR products were visualized on 1% agarose gels. Successful PCR amplifications were sent off to Epoch Life Science (Missouri City, TX, USA) for sequencing in both directions. Sequence data were visualized and edited in Geneious Prime 2020.1.2 (Biomatters Limited, Auckland, New Zealand) to generate consensus sequence per sample per locus. New sequences were deposited on GenBank under the accession codes listed in S1 Table. Additional sequences were obtained from [61]) and the NCBI GeneBank, as described above and listed in S1 Table.

### 2.3 Phylogenetic analyses

All analyses were conducted on alignments generated with MAFFT 7.450 [62] with default parameters as implemented in Geneious. Basic sequence statistics and alignment characteristics were assessed in Geneious. Nucleotide diversity among *Porites* samples per marker and across markers was calculated with MEGA X [63].

For phylogenetic analyses, nucleotide positions of ambiguous homology were removed using GBlocks 0.91b [64]. Each genetic locus was processed individually using default parameters, except for gap positions that were allowed if present in less than half of all samples. All four loci were then concatenated into one alignment using Geneious. Pre- and post-GBlocks alignment lengths for each locus and the concatenated alignment are listed in Table 1.

**Table 1:** Overview of the analyzed dataset, including sequencing and phylogenetic statistics for each marker and the entire dataset.

Marker	MT09	MT12	MT16	MT20	Overall
Full alignment length [bp]	801	901	916	1,077	3,695
Non-coding regions [bp]	138	75	35	122	370
	(17.2%)	(8.7%)	(3.8%)	(11.3%)	(10.0%)
Coding regions [bp]	663	826	881	955	3325
	(82.8%)	(91.3%)	(96.2%)	(88.7%)	(90.0%)
Genes	ND6-ATP6	ND4-12S	COX3-COX2	ND5-ATP8	
*Porites* Samples	132	136	119	126	136
Parsimony Informative Sites*	26	28	25	25	104
	(3.2%)	(3.1%)	(2.7%)	(2.3%)	(2.8%)
Singletons	24	21	23	34	102
	(3.0%)	(2.3%)	(2.5%)	(3.2%)	(2.8%)
Conserved Sites	750	833	867	1018	3468
	(93.6%)	(92.5%)	(94.7%)	(94.5%)	(93.9%)
Absolute Nucleotide Diversity (S.E.)	2.7	3.3	3.4	3.6	11.6
	(0.6)	(0.7)	(0.9)	(0.9)	(1.2)
Relative Nucleotide Diversity (S.E.)	0.0032	0.0031	0.0035	0.0032	0.0029
	(0.0007)	(0.0007)	(0.0009)	(0.0008)	(0.0003)
Length post G-blocks [bp]	766	881	915	1,076	3,638
	(95.6%)	(97.8%)	(99.9%)	(99.9%)	(98.5%)

*Among *Porites* species

Phylogenetic analyses were conducted using Bayesian inference and maximum likelihood. IQ-TREE 2.0.5 [65, 66] was used for maximum likelihood analyses including model selection [67] for each individual locus, for all four loci combined and for the proposed barcoding combination MT12-MT20 [68]. For each individual locus, the best model according to the Bayesian Information Criterion was GTR+F+I+G4 but in the partitioned analyses with all four loci, the best model was K3Pu+F+G4 (BIC score: 19,977; LnL: -9034, df:233). Node support was assessed with 10,000 ultrafast bootstrap replications [69], 10,000 SH approximate likelihood ratio test replicates and 10,000 approximate Bayes tests [70].

In addition, Maximum likelihood analyses of individual loci and the concatenated 4-loci alignment were conducted using RAxML 8.2.8 on XSEDE [71] as implemented on the CIPRES web portal [72]. A unique GTR model of sequence evolution was specified for each partition, i.e. each locus, with corrections for a discrete gamma distribution (G) for site-rate heterogeneity (GTRGAMMA). Nodal support was estimated via 1000 rapid bootstraps [73].

Bayesian inference analyses were carried out with MrBayes 3.2 [74]. Analyses were conducted with three different models of sequence evolution: The default model (nset = 0), the GTR model (nset = 6) selected by IQ-TREE and the HKY model (nset = 2) suggested by jModelTest [75] under the Bayesian information criterion (BIC). Gamma corrections and a proportion of invariable sites were included in all three models. MrBayes analyses started with random trees, default priors and two runs, each with 4 Markov chains. Convergence diagnostics were analyzed using the *sump* command as implemented in MrBayes and Tracer 1.7 [76]. The runs were allowed to proceed until the average deviation of split frequencies reached <0.01 (~1.5 M generations). Phylogenetic trees were summarized as a 50% majority-rule consensus tree with a burn-in of 25% removed using the *sumt* command as implemented in MrBayes.

### 2.4 Morphospecies determination

Species determinations for the Guam specimens were made using a combination of sources, including the original species descriptions, Veron and Pichon [77], Veron et al. [78], and unpublished species descriptions provided by the late Richard Randall. Determinations were primarily based on a qualitative assessment of colony and corallite morphology with corallite diameter measurements used to assist in assigning determinations for several of the Guam specimens.

The use of "cf." for most of the species determinations indicates uncertainty in species identification based on qualitative morphological assessment. This uncertainty is a result of slight differences between the morphology of Guam specimens and that reported in the original descriptions, the limited utility of some of the original descriptions, inconsistencies between authors, the lack of high-resolution images for type material, the lack of available genetic data for type material (or from topotypes), challenges inherent in assessing members of the genus *Porites* (e.g., the reliance of traditional morphology-based taxonomy on relatively subtle, qualitative differences in corallite characters; the sometimes high degree of intracolonial variability in corallite morphology; the lack of comprehensive morpho-molecular studies that identify morphological characters that reliably align with the molecular data), or a combination of all of these factors.

Even those determinations for which “cf.” was not used (e.g., *P*. *rus*, *P*. *lutea*, *P*. *cylindrica*) should be considered as at least somewhat tentative. For example, while the colony and corallite morphology of the Guam *P*. *lutea* specimens were highly similar to that described by Milne Edwards & Haime (1851) and several subsequent authors, and formed a clade with a specimen from Hainan, China, for which the whole mitogenome was published by Niu et al. [79], our study did not include *P*. *lutea* material from the type locality (Fiji), and we did not examine the voucher material associated with the published *P*. *lutea* mitogenome. Similarly, our study did not include *P*. *cylindrica* or *P*. *rus* material from the type localities. The species names provided below are meant to provide readers with the names of one or more described species to which we believe the specimen most closely compares. The use of the qualifier “-like” in the clade names is meant to convey the doubt regarding the application of species names to individual specimens within a clade, which was sometimes compounded by the inclusion of two or more morphospecies within the same clade.

## 3. Results & discussion

### 3.1 Basic results

The complete dataset for this study contained 143 samples, including 136 *Porites* specimens and 7 outgroup taxa. New sequence data for 67 *Porites* samples from Guam was generated and combined with previously published sequences for 69 specimens, mostly from Hawaii (S1 Table). For each specimen, sequencing data for 2–4 mitochondrial markers was analyzed with an average coverage of 3.8 markers/sample. For each marker, on average 128 *Porites* samples were analyzed on average.

Markers consisted of 800–1,100 nucleotides for a complete alignment of 3,695 nucleotides across all four markers (Table 1). This final alignment contained 104 parsimony informative sites across the 136 *Porites* specimens (PIS, 2.8%), which were fairly evenly distributed across all four markers (Table 1), and 3468 invariable sites (94%). The absolute nucleotide diversity was 11.6 base differences per sequence and the relative nucleotide diversity was 0.0029 base substitutions per site. Both types of nucleotide diversity varied little among markers, with slightly more diversity in MT16 and MT20 compared to MT09 and MT12 (Table 1).

Alignment curation with G-blocks removed 57 ambiguous nucleotide positions across all 4 markers (1.5%), predominantly in markers MT09 (n = 35) and MT12 (20), for a final sequence alignment of 3638 bp (Table 1). ModelTest with IQ-TREE identified GTR as the most suitable model for all individual markers but a K3Pu model across all 4 markers. In contrast, the stand-alone version of modelTest suggested HKY for individual markers and a TVM model across all 4 markers.

### 3.2 Phylogenetic results

**Fig 1:**
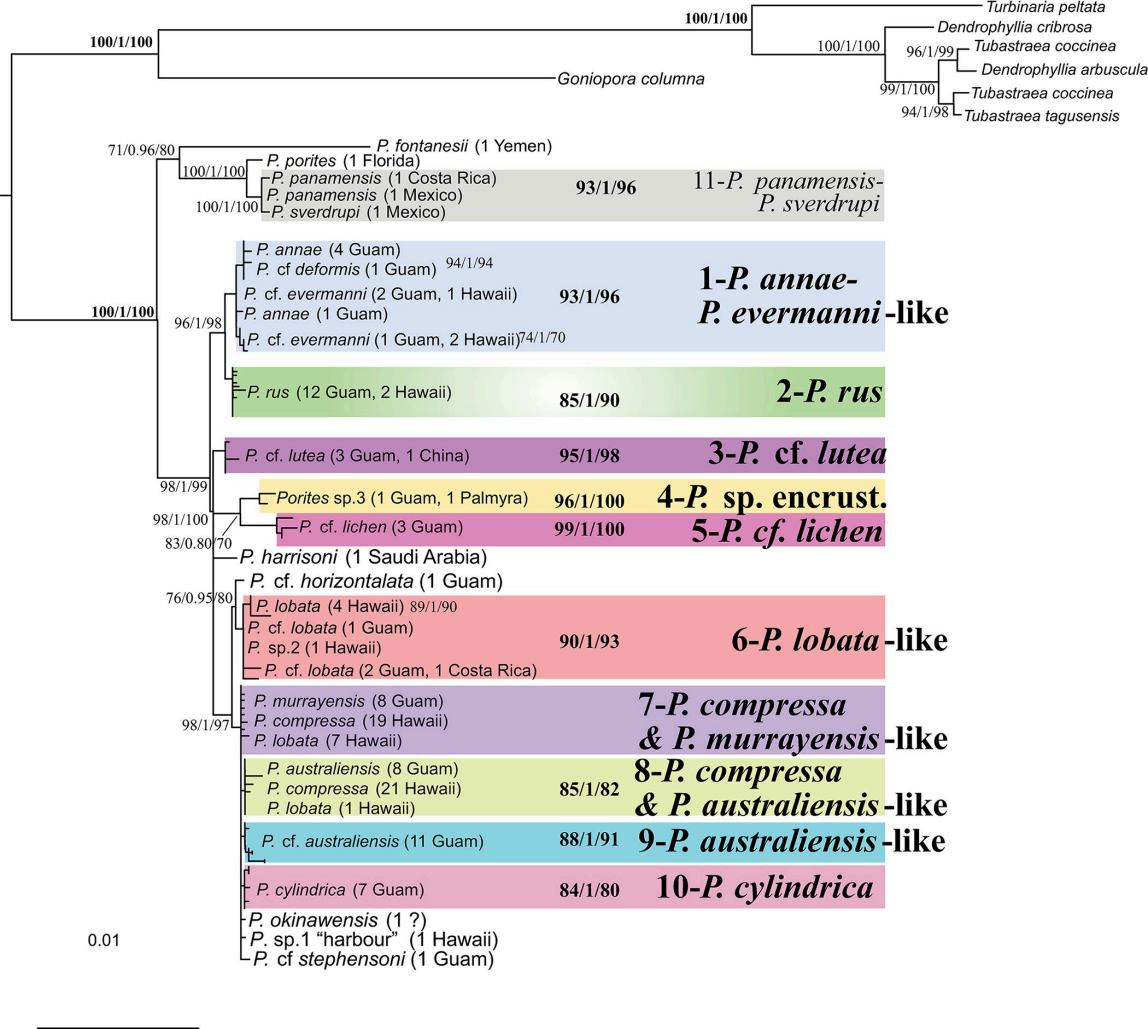
Phylogenetic relationships of *Porites* corals based on maximum-likelihood analysis of all four concatenated mitochondrial markers (3638bp post G-blocks) with IQ-TREE (lnL = -9023).

All three phylogenetic algorithms generated nearly identical results with the same overall topology and similar node support. The only notable difference was the presence of Clade 9 in both Maximum Likelihood (ML) analyses (Fig 1 & S1 Fig), which was absent from the Bayesian approximation (BA) tree generated with MrBayes (S2 Fig).

This tree is based on the K3Pu+F+G4 model of sequence evolution, as identified by IQ-TREE after merging all four loci into one partition to increase model fit (BIC score: 19,958). RAxML and MrBayes analyses resulted in a similar tree with identical overall topology and identical clade composition, with the exception of clade 9 (S1 & S2 Figs; see methods and results for further details). Numbers on nodes indicate ultrafast bootstrap support percentages based on 10,000 replications, 10,000 SH approximate likelihood ratio test replicates and 10,000 approximate Bayes tests as calculated with IQ-TREE. Bold numbers in clades indicate node support statistics for each clade.

The ML analysis with IQ-TREE identified a final tree (Fig 1) with a log-likelihood of “-9023”. This tree is based on the K3Pu+F+G4 model of sequence evolution, as identified by IQ-TREE after merging all four loci into one partition to increase model fit (BIC score: 19,958). The ML analysis with RAxML based on a GTR model of sequence evolution for each locus partition generated a virtually identical phylogenetic tree with a log-likelihood of “-9006” (S1 Fig). Bayesian analyses with MrBayes using different models of sequence evolution (HKY, F81 and GTR as proposed by ModelTest and IQ-TREE) for all marker partitions resulted in identical tree topologies with very similar node support values. Trees obtained from MrBayes all had a similar overall topology, compared to the Maximum Likelihood trees, with the mentioned exception of combining clades 7 and 9 (S2 Fig)—despite a consistent nucleotide difference in marker MT20, which was available for 9 out of 11 Clade 9 samples and 30/34 Clade 7 samples.

The monophyly of the genus *Porites* was supported in all analyses with perfect node support, i.e. >99% bootstrap support (BS) and >0.99 Bayesian probability (BP). Many nominal morphospecies were recovered as monophyletic clades with moderate to high node support, for example *Porites rus* (Forskål, 1775), *P*. cf. *lutea* *Porites lutea* Milne Edwards & Haime, 1851, *P*. cf. *lichen* (Dana, 1846) and *P*. *cylindrica* Dana, 1846. Several other monophyletic clades contained more than one traditional species, which might be due to a lack of resolution among mitochondrial markers or due to phenotypic plasticity or hybridization as discussed in detail below.

The most basal split among *Porites* species separates a group of species from the Caribbean (*P*. *porites* (Pallas, 1766)), Tropical Eastern Pacific (TEP, *P*. *panamensis* Verrill, 1866, *P*. *sverdrupi* Durham, 1947), and Red Sea (*P*. *fontanesii* Benzoni & Stefani, 2012) from the remaining Central West Pacific (CWP) *Porites* species. This basal split has been observed before [60] and is confirmed here with strong node support. Within this basal group, *P*. *fontanesii* is sister to the remaining samples and particularly distinct compared to all other samples (Table 2), which matches its geographic isolation in this dataset. In the Caribbean and Tropical Eastern Pacific clade, the three specimens of *P*. *panamensis* (n = 2) and *P*. *sverdrupi* (1) from the Tropical Eastern Pacific clustered together to form Clade 11. This clade has been recognized by Forsman et al [60] as well, with identical species composition and is termed clade XI there (Table 3).

**Table 2:** Overview of the 11 well-represented clades and the 7 distinct morphospecies, including the node support for each clade in each phylogenetic analyses and the number of nucleotides that supports each clade in each individual marker, the number of markers and nucleotides overall that support each clade. The last column indicates the number of species names of all morphospecies identified within each clade.

Clade	Samples	Clade support			Barcode suitability						Morphospecies	
		IQ- TREE	RAxML	Mr Bayes	MT09	MT12	MT16	MT20	# Loci	BP		
**Main species, well-represented here:**												
**1-*P*.*annae- P*.*evermanni***	**12**	**93**	**87**	**100**	**1**	**1**	**-**	**1**	**3**	**3**	**3**	***P*. *annae*,** ***P*. cf. *evermanni*,** ***P*. cf. *deformis***
**2-*P*.*rus***	**14**	**85**	**75**	**91**	**1**	**1**	**-**	**1**	**3**	**3**	**1**	***P*. *rus***
**3-*P*.cf.*lutea***	**4**	**95**	**94**	**100**	**1**	**1**	**1**	**1**	**3**	**3**	**1**	***P*. *lutea***
**4-*P*.sp.encrust**	**2**	**96**	**100**	**100**	**2**	**1**	**1**	**2**	**4**	**6**	**1**	***P*. sp.**
**5-*P*.cf.*lichen***	**3**	**99**	**100**	**100**	**3**	**3**	**3**	**1**	**4**	**10**	**1**	***P*. cf. *lichen***
**6-*P*.*lobata*-like**	**9**	**90**	**84**	**100**	**-**	**2**	**1**	**1**	**3**	**4**	**2**	***P*. cf. *lobata***
												*P*. sp.2 (Hawaii, 1)
**7-*P*.*compressa-*** ***P*.*murrayensis-*** **like**	**34**	*87	*94	*94	**-**	**-**	**-**	**-**	0	0	**3**	***P*. cf. *murrayensis*,** ***P*. *compressa*** *P*. *lobata* (Hawaii, 1)
**8-*P*.*compressa-*** ***P*.*australiensis*-** **like**	**30**	**85**	**50**	**63**	**-**	**1**	**-**	**-**	1	1	**2**	***P*. cf. *australiensis*,** ***P*. *compressa***
**9*-P*.*australiensis*-** **like**	**11**	**88**	**16**	**-**	**-**	**-**	**-**	**1**	1	1	**1**	***P*. cf. *australiensis***
**10-*P*.*cylindrica***	**7**	**84**	**55**	**67**	**1**	**-**	**-**	**-**	1	1	**1**	***P*. *cylindrica***
11*-P*.*panamensis-* *P*.*sverdrupi*	3	91	99	100	1	1	1	2	4	5	2	*P*. *panamensis*, *P*. *sverdrupi*
Minor species, i.e. not well-represented here:												
*12-P*.*harrisoni*	1				2	2	2	1	**4**	**7**	1	*P*. *harrisoni*
*13-P*.*porites*	1				1	1	1	1	**4**	**4**	1	*P*. *porites*
*14-P*.*fontanesi*	1				>3	>3	>3	>3	**4**	**>12**	1	*P*. *fontanesii*
*15-P*.cf. *horizontalata*	1				1	1	1		**3**	**3**	1	*P*. cf. *horizontalata*
*16-P*.cf.*stephensoni*	1				-	-	2		1	2	1	*P*. cf. *stephensoni*
*17-P*.sp.harbour	1				-	-	-	1	1	1	1	*P*. sp.1 harbour
*18-P*.*okinawaensis*	1				1	-	-	-	1	1	1	*P*. *okinawaensis*

**Table 3:** Overview of the correspondence of phylogenetic clades identified in this study with clades reported in other phylogenetic studies of *Porites* corals as well as the morphospecies reported for each clade in these studies.

This study	Morphospecies		Forsman et al 2009		Forsman et al 2020		Terraneo et al 2021		Primov et al. in prep	
**1-*P*.*annae-*** ***P*.*evermanni*-like**	3	*P*. *annae*, *P*. cf. *evermanni*, *P*. cf. *deformis*	II	*P*. *annae* *P*. *evermanni*	-	-	-	-	I	*P*. *evermanni*
**2-*P*.*rus***	1	*P*. *rus*	II	*P*. *rus*, *P*. *monticulosa*	-	-	IV	*P*. *rus*, *P*. *monticulosa*	-	*-*
**3-*P*.*cf*.*lutea***	1	*P*. *cf*. *lutea*	V	*P*. *lutea*, *P*. *lobata*	-	-	VIII	*P*. *somaliensis*	II	*P*. cf. *lutea*
**4-*P*.sp.encrust.**	1	*P*. sp. encrust,	-	-		^1^*P*. sp3	-	-	-	*-*
**5-*P*.cf.*lichen***	1	*P*. cf. *lichen*	VIII	*P*. *lichen*	-	-	-	-	-	*-*
**6-*P*.*lobata*-like**	1	*P*. *cf*. *lobata*	I	Many *Porites* spp.	A	*P*. *lobata*	V	Many *Porites* spp.	IV	*P*. *cf*. *lobata*
**7-*P*.*compressa- P*.*murrayensis-* like**	3	*P*. cf. *murrayensis*, *P*. *compressa* *(P*. *lobata* HI*)*	I	Many *Porites* spp.	B	*P*. *lobata*, *P*. *compressa*	V	Many *Porites* spp.	V	*P*. cf. *murrayensis*
**8-*P*.*compressa*- *P*.*australiensis-* like**	2	*P*. cf. *australiensis*, *P*. *compressa*	I	Many *Porites* spp.	C	*P*. *compressa*, *P*. cf. *lobata*	V	Many *Porites* spp.	VII	*P*. cf. *australiensis*
**9*-P*.*australiensis*- like**	1	*P*. cf. *australiensis*	I	Many *Porites* spp.	-	-	V	Many *Porites* spp.	VI	*P*. cf. *australiensis*
**10-*P*.*cylindrica***	1	*P*. *cylindrica*	I	Many *Porites* spp.	-	-	V	Many *Porites* spp.	-	*-*
11-*P*.*panamensis -P*.*sverdrupi*	2	*P*. *panamensis*, *P*. *sverdrupi*	XI	*P*. *panamensis*, *P*. *sverdrupi*	-	-	-	-	-	-
12-*P*.*harrisoni*	1	*P*. *harrisoni*	I	Many *Porites* spp.	-	-	V	Many *Porites* spp.	-	-
13-*P*.*porites*	1	*P*. *porites*	X	*P*. *furcata* *P*. *divaricata*	-	-	-	-	-	-
14-*P*.*fontanesi*	1	*P*. *fontanesi*	-	-	-	-	I	*P*. *fontanesi*	*-*	*-*
15-*P*.*cf*. *horizontalata*	1	*P*. cf. *horizontalata*	-	-	-	-	-	*-*	*-*	*-*
16-*P*.*cf*. *stephensoni*	1	*P*. cf. *stephensoni*	-	-	-	-	-	*-*	*-*	*-*
17-*P*.sp.harbour	1	*P*. *harbour*	-	-	^2^	*P*. sp1	-	*-*	*-*	*-*

^1,2^ Both samples were identified as unique and significantly different in phylogenetic analyses based on mitochondrial genomes as well as 3 million SNPs derived via ezRAD in Forsman et al 2020 [61].

^3^Primov et al (in prep) refers to a recent phylogenomic study of massive *Porites* corals based on samples collected in different reef habitats around Guam, which is currently in preparation for publication. A ddRAD approach was used there to a generate genome-wide dataset for massive *Porites* corals that was analyzed with multiple phylogenetic approaches, which led to a well-supported phylogenetic tree with distinct monophyletic clades that corresponds to Clades 1, 3, 6, 7, 8, and 9, as outlined in this table.

The remaining, predominantly Indo-West Pacific *Porites* samples fall into two separate, well-supported and clearly distinct clades. The first clade contains two subclades:

**Clade 1** is well-supported (node support 93/87/100 in IQ-TREE, RAxML and MrBayes) and contains all 5 samples identified as *P*. *annae* Crossland, 1952 from Guam, the only *P*. cf. *deformis* Nemenzo, 1955 specimen, and all 6 samples identified as *P*. *evermanni* Vaughan, 1907 from Hawaii and Guam. This clade is defined by clade-specific mutations in three of the four analyzed markers (MT09, MT12 and MT20, Table 3). Clade 1 corresponds to Clade II in Forsman et al [60], where it contained 6/6 *P*. *annae* from Samoa and 1/3 *P*. *annae* from Hawaii, all *P*. *evermanni* from Hawaii and several unidentified *Porites* sp. from Hawaii and Panama. These species encompass significantly different and distinct morphologies, from massive *P*. *evermanni* to branching/columnar *P*. *annae* and *P*. *cf*. *deformis*. Using scanning electron microscopy, Forsman et al [60] noted in this clade, the corallite “walls, denticles, palli, and columella tended to have similar height, with more evenly spaced and deeper interstitial holes” than in other clades. This clade was also recovered in multi-marker phylogenetic analyses by Hellberg et al [24]. A recent phylogenomic study of massive *Porites* on Guam (Primov et al, in prep) recovered this clade as well and termed it clade I. The clade was absent in recent *Porites* studies from the Arabian Peninsula [50, 80], indicating that it may be a predominantly Pacific clade, where it is widespread, from the Tropical Eastern Pacific, across the Central, West and South Pacific.

**Clade 2** is mostly well supported (85/75/91) and contains all 14 samples identified as *P*. *rus* from Guam (12 specimen), Hawaii (1) and an unknown location (from NCBI, 1). This clade is distinguished by clade-specific mutations in three out of four markers as well (MT09, MT12 and MT20, Table 3). Our Clade 2 corresponds to Clade III in Forsman et al ([60]) and Clade IV in [50, 80], where it contained *P*. *rus* from Hawaii, Tahiti and the Arabian Peninsula as well as the morphologically similar *P*. *monticulosa* Dana, 1846 from Hawaii and the Arabian Peninsula. Despite this enormous geographic range, this clade is among the most morphologically uniform and phylogenetically distinct clades of *Porites* in recent studies. The monophyly and species composition of this clade was further confirmed by Hellberg et al [24].

The second major Indo-West Pacific clade is more complex, starting with an unresolved polytomy (with IQ-TREE, RAxML and MrBayes) between the only *P*. *harrisoni* Veron, 2000 sample in this study from a mitochondrial reference genome (NC037435) originally collected in Saudi Arabia with seven private mutations across all 4 markers (Table 3), as well as three distinct, generally well-supported clades:

**Clade 3** (95/94/100) contains a *P*. *lutea* mitochondrial reference genome (KU159432) from Hainan Island, China, as well as three *P*. *cf*. *lutea*-like samples from Guam. It is distinguished by clade-specific mutations in all four markers (Table 3). Our Clade 3 most likely corresponds to Clade V in Forsman et al. [60], which contains most *P*. *cf*. *lutea* as well as some *P*. *lobata* Dana, 1846 from Samoa and has a very similar phylogenetic position. It would then also correspond to Clade VIII in Terraneo et al [50], where it was represented by colonies identified as *P*. *somaliensis* Gravier, 1910 from Djibouti, Yemen and Saudi Arabia. Interestingly, Veron [16] states that the corallites of *P*. *somaliensis* are in fact most similar to *P*. *lutea*, which further testifies to the correspondence of these clades. Comparisons with Hellberg et al [24] are difficult here since the few *P*. *lutea* specimens were spread rather widely across their phylogenies.

Another well-supported clade (94/85/100) contains two subclades, termed Clade 4 and Clade 5 here. **Clade 4** (96/100/100) consists of two morphologically similar, encrusting *Porites* specimens: a potentially undescribed species from Guam (*P*. *cf*. *vaughani* Crossland, 1952; BP13, UOG IZC #14) and a specimen identified as *P*. *superfusa* Gardiner, 1898 by Forsman et al [60] from Palmyra atoll (NCBI: SAMN06648869). It is distinguished by 6 clade-specific mutations across all four markers (Table 3). This clade was not previously reported by Forsman et al. [60] or Terraneo et al [50]. While the Palmyra specimen was identified as *P*. *superfusa*, the relatively large mammiform surface swellings and superficial calices visible in the small in situ image of the specimen in Forsman et al. [81] are more consistent with the undescribed species from the Marianas than with Gardiner’s *P*. *superfusa* description from Tuvalu. However, an examination of the skeletal material for both specimens, and ideally genetic data from the type localities would be required for proper comparison. **Clade 5** (99/100/100) contains all three *P*. cf. *lichen* samples from Guam and is distinguished by ten clade-specific mutations across all four markers (Table 3). This clade corresponds to Clade VIII in Forsman et al. [60], which contains all seven *P*. *lichen* present in that study. It was not recorded by Terraneo et al [50] around the Arabian Peninsula.

The next clade (89/94/100) contains five nested subclades, which were not distinguished by Forsman et al. [60] or Terraneo et al. [50], where this undivided clade is termed Clade I and Clade V, respectively. Here, these subclades are distinguished due to shared unique mutations across enormous geographic distances, moderate to high node support and distinct, clade-specific morphologies (Fig 1, Table 2), which merit their consideration as distinct evolutionary units. Moreover, a recent ezRAD study by Forsman et al [61] in Hawaii distinguished 3 of these clades (Clades 6, 7 and 8) while the other two are likely absent in Hawaii and around the Arabian Peninsula.

The most basal split separates a clade (77/77/98) containing a well-supported subclade, termed Clade 6, as well as the only *P*. cf. *horizontalata* Hoffmeister, 1925 specimen from Guam. The single *Porites* cf. *horizontalata* colony presented in this study was composed of encrusting laminae and irregular branches, with small corallites similar to that of *P*. *rus*. The sample contains individual and/or species-specific mutations in 3 out of the 4 tested mitochondrial markers (MT09, 12 & 16; Table 3). It is interesting to note that the *P*. cf. *horizontalata* specimen here is only distantly related to *P*. *rus*, despite exhibiting a similar colony and corallite morphological characters. **Clade 6** (90/84/100) is similarly distinguished by four clade-specific mutations across 3 markers (MT12, MT16 & MT20; Table 3). It contains a *P*. *lobata* specimen from Costa Rica, three *P*. cf. *lobata* from Guam, and four *P*. *lobata* and one *Porites* sp. from Hawaii. Samples in this clade shared four clade-specific mutations between Costa Rica and Guam, i.e. over 14,000 km of open ocean. This clade is termed Clade A in Forsman et al [61] where it contains mostly *P*. *lobata*.

The clade on the other side of the basal split (87/94/94) contained a group of “basal” specimens that were not defined by mutations in any of the four analyzed mitochondrial markers. Instead, it is defined by the absence of additional differentiation compared to Clades 8–10. This group is comprised of 8 *P*. cf. *murrayensis* Vaughan, 1918 from Guam as well as 19 *P*. *compressa* Dana, 1846 and 7 *P*. cf. *lobata* from Hawaii. Although in our phylogenetic analyses these specimens are paraphyletic and do not form a distinct monophyletic clade, this group is termed “**Clade” 7** here since it was recovered as a distinct mitochondrial Clade B in Forsman et al [61] and as Clade V in Primov et al (in prep). Moreover, specimens on Guam are morphologically distinct from other clades/species (but closest to *P*. *lobata*) and were identified as *P*. cf. *murrayensis* based on their relatively small corallites (usually <1.2 mm in diameter), thick walls, and prominent palli associated with the lateral septal pairs. Interestingly, although the Hawaii *P*. *compressa* and *P*. cf. *lobata* specimens share identical mitochondrial genomes, genome-wide ezRAD analyses were able to recover them as distinct [61]. This clade thus seems to contain distinct evolutionary units, which are resolvable with mtDNA alone—at least on Hawaii.

In addition, three samples were present at this polytomy that belong to three distinct morphospecies and contain unique individual and thus potentially species-specific mutations: a single *P*. *okinawensis* Veron, 1990 specimen with one private mutation in MT09, a single *P*. cf. *stephensoni* Crossland, 1952 specimen from Guam with two private mutations in MT16, and a single “Honolulu-harbour” *Porites* specimen (sensu [82]) with a unique mutation in MT20. The “Honolulu-harbour” specimen was found to be significantly distinct in genome-wide ezRAD analyses [61], which supports highlighting these moderately-supported single-sample species. Since only one sample of each species was available, it is not possible to distinguish individual variation from species-level distinctiveness and other samples in this study also showed elevated levels of unique variation (e.g. MZ05_Plobata).

In addition, 3 distinct subclades were identified:

**Clade 8** (85/50/63) contains 8 massive *P*. cf. *australiensis* Vaughan, 1918 from Guam as well as 21 branching *P*. *compressa* and 1 massive *P*. cf. *lobata* from Hawaii. This clade is supported by a single nucleotide change in MT12 but was also identified as a distinct clade in Forsman et al ([61] as Clade C) and Primov et al (in prep.; as Clade VII). Interestingly, while the gross colony and corallite morphology of the Guam specimens in this clade are quite similar to that of the Guam specimens in Clade 9, all of the Clade 8 specimens exhibited a brown or greenish-brown coloration, while all Clade 9 specimens were purple or purplish-brown (see e.g. Fig 3).

**Clade 9** (88/16/-) contains 11 massive *P*. cf. *australiensis*, which were all sampled from two nearby populations on the southwest coast of Guam. It is characterized by a single mutation in MT20 but was not recovered in the MrBayes analyses, independent of the selected model of sequence evolution (HKY or GTR). It was recovered as Clade VI in Primov et al (in prep.) but it is unclear if it occurs outside of Guam/the Marianas. Its morphology is similar to the closely related Clade 8, but showed a different coloration, as mentioned above.

And finally, **Clade 10** (84/50/67) contains all 7 *P*. *cylindrica* specimens from Guam. This species is branching and resembles *P*. *compressa* from Hawaii in overall colony morphology. It is genetically distinct by a single mutation in MT09. It was not included in Primov et al (in prep) or any other *Porites* phylogenetic studies to date, as far as we know.

### 3.3 Clades, species & barcodes

In total, 11 *Porites* clades were identified and are distinguished here based on a combination of node support (Table 2), morphospecies identity and comparisons with genome-wide sequence datasets [50, 61]. All 11 clades were supported by both ML analyses (Fig 1 & S1 Fig) and only one of them, Clade 9-*P*.*australiensis*-like, was not recovered in the MrBayes analyses—despite a unique mutation in marker mt20 (Fig 1, S1 & S2 Figs). Moreover, many of these clades correspond to clades identified by [24, 50, 60, 61] and Primov et al (in prep), using mitochondrial, nuclear and genomic DNA sequence markers (Table 3)–as detailed above. In addition, individual specimens of seven distinct morphospecies are highlighted here to report the potential ability of these markers to distinguish them in future studies. Additional specimens will need to be analyzed to assess if their distinctiveness is indicative for the entire species or merely represent individual variation of the sampled specimen. In general, the proposed barcode markers will all need to be tested in more species and locations to verify their general suitability.

**Fig 2:**
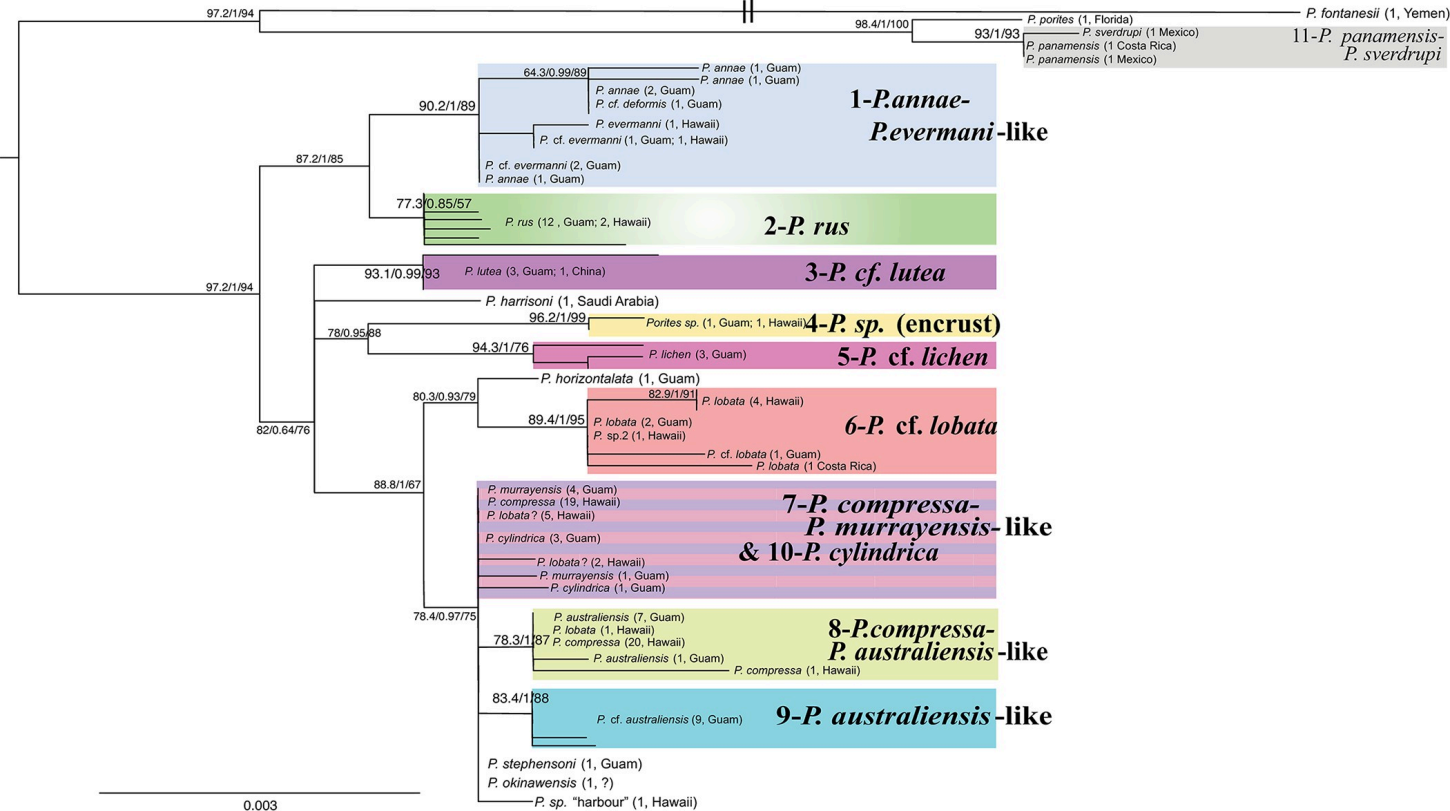
Phylogenetic relationships of *Porites* corals based on maximum-likelihood analysis of the proposed barcode markers mt12 and mt20 (1957bp) with IQ-TREE (lnL = -3503). This tree is based on the HKY+F+I model of sequence evolution, as identified by IQ-TREE after merging both loci into one partition to increase model fit. Numbers on nodes indicate ultrafast bootstrap resampling percentages based on 10,000 replications, 10,000 SH approximate likelihood ratio test replicates and 10,000 approximate Bayes tests.

Of the 11 multi-sample *Porites* clades that are distinguished here, 7 are consistently supported by all or least most of the individual mitochondrial markers (Table 2). In turn, each of the four mitochondrial barcodes was able to distinguish at least 5 well-represented clades and 10 potential species (Table 2). A combination of the two most promising markers, MT12 and MT20, distinguished 9 well-represented clades and 12 potential species overall (Fig 2). We therefore propose this combination as a suitable, low-cost barcoding approach to distinguish *Porites* species. For example, on Guam, all 11 well-represented clades can be identified with these two barcodes since *P*. *cylindrica* can be readily distinguished morphologically. In addition, this barcode combination identifies 14 of the 18 potential species and increases reliable clade identification compared to each individual marker by increasing the number of informative SNP for most clades and species (Table 2). RFLP approaches to distinguish between these clades without sequencing might be possible but were not designed or tested yet.

**Fig 3:**
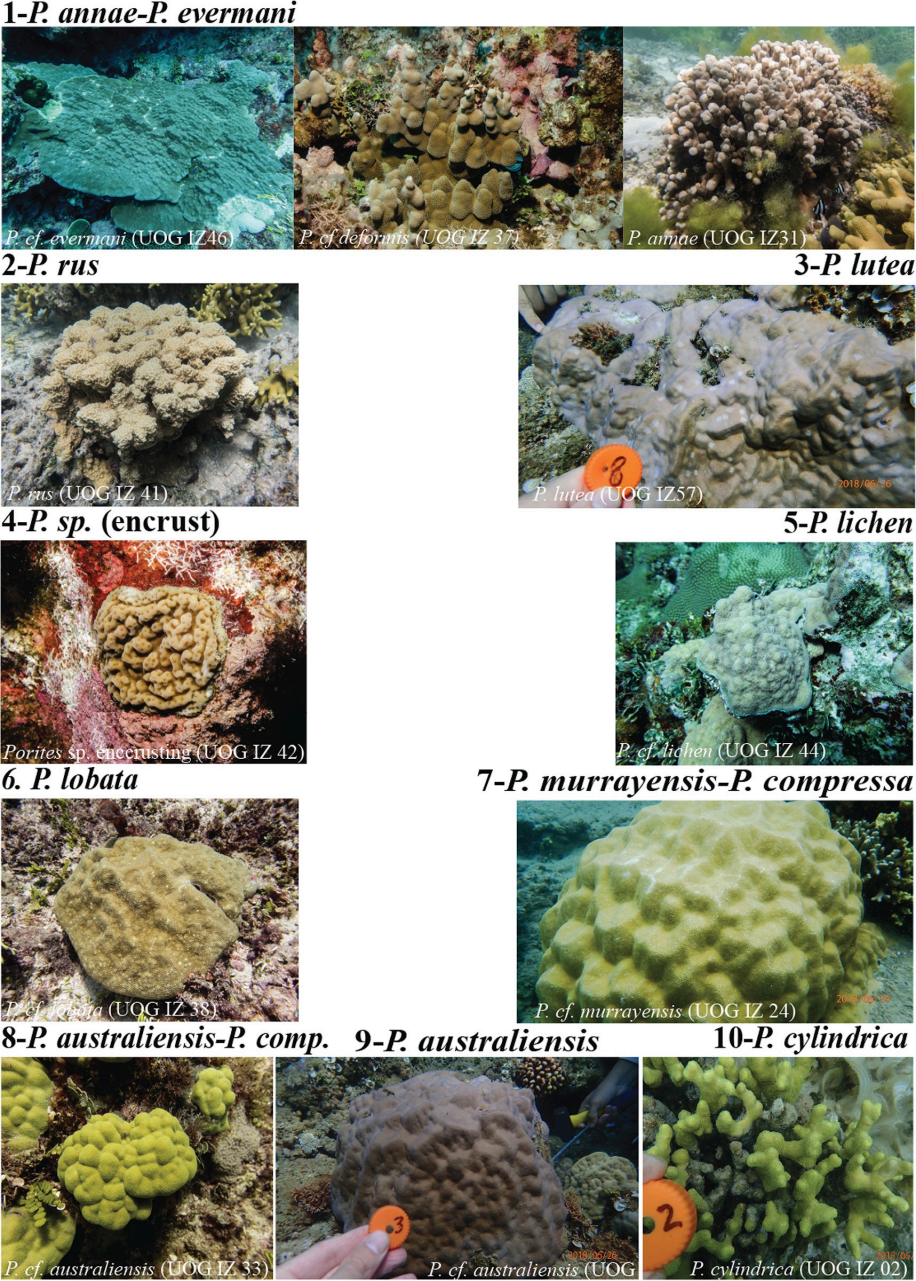
Representative morphospecies for each genetic clade.

Although there was an overall good congruence between our phylogenetic results and the samples taxonomic classification based on morphology, there were some discrepancies as well—as has been observed previously for *Porites* corals (e.g. [24, 60, 61, 81]). For once, at least three clades contain samples with distinct morphologies, i.e. more than one morphospecies (Clades 1, 7 & 8). For 2 out of these 3 clades, this is due to previous taxonomic classifications [61], which could not be verified personally. In contrast, *P*. *annae* and *P*. *evermanni* were both collected and examined on Guam. Although their colony morphology is fundamentally different (Fig 3), their micromorphological corallite structures are similar and they are genetically not clearly distinct based on the 4 mitochondrial markers analyzed here. This is most likely due to incomplete lineage sorting at the species level, which is surprising for a mitochondrial marker that should sort faster than nuclear loci. Interestingly, Forsman et al [60] found both species to be indistinguishable at the nuclear ITS2 marker as well, which indicates widespread incomplete lineage sorting, i.e. including the nuclear genome, at least in Hawaii. Since both species are widespread throughout the Indo-Pacific, recent speciation seems unlikely but their presumably large effective population sizes could slow down and drag out complete lineage sorting for a very long time. The smaller populations in semi-isolated locations like Guam and Hawaii should sort faster and there is some indication for that on the trees (Fig 1, S1 & S2 Figs), which does show two subclades, one dominated by *P*. *annae* and the other dominated by *P*. *evermanni*. Lineage sorting thus seems to be happening but has not been completed yet.

On the other hand, three morphospecies appeared in more than one clade, namely *P*. *compressa* (Clades 7 & 8), *P*. *cf*. *australiensis* (Clades 8 & 9) and *Porites cf*. *lobata*, which appeared in three distinct clades (6, 7 & 8). In the case of *P*. *cf*. *lobata*, all specimens from Guam (3) and Costa Rica (1) as well as 4 of the 12 *P*. *lobata* from Hawaii were found in Clade 6. In addition, 7 *P*. *lobata* from Hawaii were found in clade 7 and one in clade 8. Since all three clades are closely related and *Porites lobata* has an Indo-Pacific wide distribution and is abundant in many places, incomplete lineage sorting might be responsible for the ambiguous genetic identification. However, since only Hawaiian specimens occur in more than one clade and Hawaii is more isolated and presumably less diverse than Guam, incomplete lineage sorting is less likely there and introgressive hybridization could be responsible for observed ambiguity. In fact, geographically limited introgressive hybridization has been reported involving one of these *Porites* species, *P*. *lobata*, in the even more isolated Tropical Eastern Pacific [24]. Ideally, the Hawaii specimen in question should therefore be re-examined carefully, focusing on characters like the corallite diameter and palli, which we found suitable to distinguish *P*. *cf*. *lobata* from *P*. *cf*. *murrayensis* (clade 7) and *P*. *cf*. *australiensis* (clade 8).

In contrast, *P*. *compressa* specimens were almost equally split between closely related Clades 7 (19) and Clade 8 (21). This is surprising since *Porites compressa* is a local endemic in Hawaii, which makes further cryptic species less likely and should facilitates lineage sorting due to presumably smaller population sizes (although it can be abundant locally, e.g. in Kaneohe Bay; [16]). Moreover, Hellberg et al [24] found significant differences across several nuclear loci among three *P*. *compressa* samples and Forsman et al [61] found genome-wide differences between those two clades using RAD-Seq data (see Table 3), which confirms their distinctiveness despite close morphological similarities. Primov et al (in prep) further detected genome-wide differences between Guam specimens of *P*. cf. *murrayensis* and *P*. cf. *australiensis*, which represent these same two clades.

Similarly, *P*. *australiensis* is also fairly evenly split between Clade 8 (8) and Clade 9 (11). All *P*. *australiensis* specimens were collected on Guam and despite repeated micromorphological examinations, no significant differences between the two groups of *P*. *cf*. *australiensis* could be detected. Interestingly, one of these two clades was limited to the south-west corner of Guam while the other one was more wide-spread (Primov et al, in prep). The two clades with *P*. cf. *australiensis* are closely related but both have clade-specific mutations in mt12 (Clade 8) and mt20 (Clade 9). *Porites australiensis* is common and widespread across the Indo-West Pacific [16] so incomplete lineage sorting is more likely for this species. It would be surprising, however, that two distinct lineages within the same species are present on a small and remote Pacific island like Guam. Moreover, a recent RAD-Seq study found genome-wide differences between *P*. cf. *australiensis* specimens from Guam, representing Clades 8 and 9 (Primov et al., in prep). Morphological stasis in two widespread and distinct species thus seems to be the most likely explanation for the congruence of morphologies in Clades 8 and 9.

Mitochondrial markers only provide a limited and somewhat particular view of genetic differences between species and specimens. It is therefore entirely possible that the proposed markers cannot fully resolve all actual *Porites* species boundaries. Compared to previous mitochondrial barcodes for *Porites*, most major clades were also recognized using the putative control regions in Forsman et al [60] and Terraneo et al [83]. In a broadly comparable dataset from the Arabian peninsula [83], the putative control region had a slightly higher number of parsimoniously informative sites (40 vs 25–28) in a longer sequencing product (1287bp). However, three of the four tested markers (MT09, MT12 and MT20) provide some resolution for Clades 6–10, which were lumped in previous studies, based e.g. on this control region marker [60, 83]. Since different species and geographic regions were analyzed in different studies, the observed increased resolution here could also be due to the absence of some of these clades in other studies. Ultimately, only a comparison with the same samples would provide a definite answer and no mitochondrial marker was or will be able to reliably distinguish *P*. *compressa* and *P*. cf. *lobata* on Hawaii, where they shared 100% identical mitochondrial genomes [61].

## 4. Conclusion

This study provides another perspective of the species’ diversity and phylogeny in the major reef-building coral genus *Porites*. Like previous studies (e.g. [24, 50, 60, 61, 81], we found some disagreements between morphology-based species designations and genetically derived clades. However, unlike other studies, we found significantly more congruence between genetic clades and micro-morphology based taxonomy, in line with more recent phylogenomic studies of *Porites* [50, 61]. While this study did not include a quantitative analysis of morphological characters, our results suggest that micromorphology will play an important role in resolving species boundaries in *Porites*, as it did in several other scleractinian genera [21, 23, 80, 84–88]. Our results also attest to the suitability of “reverse taxonomy” approaches [31] in identifying suitable characters to distinguish between genetically-identified evolutionary species. Lastly, our results support the use of carefully-tested mitochondrial sequence data to distinguish among scleractinian species, including *Porites*, despite conflicting results in other studies. One reason might be that we used multiple mtDNA markers since no single marker was able to resolve the complex, mostly massive *Porites* species (i.e. Clades 6–10). It is also important to note that our dataset was geographically and taxonomically limited and not all clades were properly vetted using genome-wide sequence data (since no comprehensive genome-wide dataset is currently available for *Porites* corals). Mito-nuclear discordances have been observed among Porites corals before and were recently documented to be widespread among Scleractinian corals [51]. However, *Porites* corals were among the least affected and only one mismatch was documented for *P*. *cf*. *australiensis*, which is split here between clades 8 and 9. More instances of discordance will likely be discovered over time due to incomplete lineage sorting or introgression, but this study shows that mitochondrial sequencer markers are nonetheless very useful for taxonomic assessments of *Porites* species.

The generation of genome-wide sequence data for phylogenetic studies is an important next step that has recently been applied to *Porites* corals successfully around the Arabian Peninsula [50], Hawaii [57] and Guam (Primov, in prep). Since different genome subsampling techniques were used to generate genome-wide dataset for these different studies, it will be challenging to combine them in one comprehensive analysis. However, we are working with multiple groups to address these phylogenetic questions on a larger scale with more data and better phylogenetic resolution and are active looking for additional collaborators.

Quantitative morphological analyses are further required to advance our understanding of *Porites* species diversity and identification. The results of such analyses would complement phylogenetic studies like this one to arrive at sets of morphological characters used to reliably delineate molecular clades, as proposed by reverse taxonomic approaches. It is also necessary to designate representative individuals for each genetic clade to facilitate comparisons among allopatric clades and across studies. Our Table 3 is another such attempt to connect with similar studies in other geographic regions and facilitate comparisons and connections. In addition, we are generating vouchers samples, permanently stored at the University of Guam Biorepository (https://specifyportal.uog.edu), including high-resolution images of micromorphological characters, DNA sequence data (as well as primers, S2 Table, and PCR protocols etc.) for the mitochondrial barcodes proposed here and links to genome-wide datasets as we generate them. Finally, we agree with Bonito et al. [89] that an attempt to collect topotypes for all nominal species should be made, and the associated morphological and molecular data be shared, in order to facilitate the proper application of *Porites* nomenclature to the clades that emerge from integrative morpho-molecular analyses.

## Supporting information

S1 Table Sample list. (DOCX)

S2 Table Primers. (DOCX)

S1 Fig RAxML tree. (TIF)

S2 Fig MrBayes tree. (TIF)
