# Supplementary material for: Barcoding and mitochondrial phylogenetics of Porites corals
Source: PLoS One. 2024 Feb 15;19(2):e0290505. doi: 10.1371/journal.pone.0290505 (PMC10868756; doi:10.1371/journal.pone.0290505)
Supplement: S1 Table — (DOCX) [file pone.0290505.s001.docx]

### S1 Table: Sample list

List of samples and molecular markers. Guam samples were collected, amplified and sequenced by the authors and additional information about each specimen can be found at<https://specifyportal.uog.edu/>. All other sequences were extracted from mitochondrial genomes obtained from *Forsman et al (2020)* and NCBI (https://www.ncbi.nlm.nih.gov/).

| **#** | **Sample** | **Species** | **Clade** |  | **Source** | | **Sampling location** | **Marker presence** | | | |
| --- | --- | --- | --- | --- | --- | --- | --- | --- | --- | --- | --- |
|  |  | Initial morphospecies designation |  |  | Collection | Catalog # |  | MT09 | MT12 | MT16 | MT20 |
| **Guam:** | |  |  |  |  |  |  |  |  |  |  |
| 1 | BP01 | *Porites rus* | 2-P.rus |  | UOG Biorepository | IZ00030 | Guam, Pago Bay | OR509221 | OR509158 | OR509108 | OR509052 |
| 2 | BP02 | *P. annae* | 1-P.annae-   P.evermanni-like |  | UOG Biorepository | IZ00031 | Guam, Cocos Lagoon | OR509222 | OR509159 | OR509109 | OR509053 |
| 3 | BP03 | *P. cf. australiensis* | 8-P.compressa-   P.australiensis-like |  | UOG Biorepository | IZ00032 | Guam, Pago Bay | OR509223 | OR509160 | OR509110 | OR509054 |
| 4 | BP04 | *P. cf. australiensis* | 8-P.compressa-   P.australiensis-like |  | UOG Biorepository | IZ00033 | Guam, Pago Bay | OR509224 | OR509161 | OR509111 | OR509055 |
| 5 | BP05 | *P. cf. lichen* | 5-P.cf.lichen |  | UOG Biorepository | IZ00034 | Guam, Pago Bay | OR509225 | OR509162 | 0 | OR509056 |
| 6 | BP06 | *P. cf. murrayensis* | 7-P.compressa-   P.murrayensis-like |  | UOG Biorepository | IZ00035 | Guam, Pago Bay | OR509226 | OR509163 | OR509112 | 0 |
| 7 | BP07 | *P. cf. horizontalata* | 13-P.cf. horizontalata |  | UOG Biorepository | IZ00036 | Guam, Apra Harbor | OR509227 | OR509164 | OR509113 | OR509057 |
| 8 | BP08 | *P. cf. deformis* | 1-P.annae-   P.evermanni-like |  | UOG Biorepository | IZ00037 | Guam, Agat | OR509228 | OR509165 | OR509114 | OR509058 |
| 9 | BP09 | *P. cf. lobata* | 6-P.lobata-like |  | UOG Biorepository | IZ00038 | Guam, Pago Bay | OR509229 | OR509166 | OR509115 | OR509059 |
| 10 | BP10 | *P. cf. stephensoni* | 16-P.stephensoni |  | UOG Biorepository | IZ00039 | Guam, Apra Harbor | OR509230 | OR509167 | OR509116 | OR509060 |
| 11 | BP11 | *P. cylindrica* | 10-P.cylindrica |  | UOG Biorepository | IZ00040 | Guam, Cocos Lagoon | OR509231 | OR509168 | 0 | OR509061 |
| 12 | BP12 | *P. rus* | 2-P.rus |  | UOG Biorepository | IZ00041 | Guam, Cocos Lagoon | OR509232 | OR509169 | OR509117 | OR509062 |
| 13 | BP13 | *P. cf. vaughani* | 4-P.sp.encrusting |  | UOG Biorepository | IZ00042 | Guam, Agat | OR509233 | OR509170 | OR509118 | OR509063 |
| 14 | BP14 | *P. cf. lichen* | 5-P.cf.lichen |  | UOG Biorepository | IZ00043 | Guam, Pago Bay | OR509234 | OR509171 | OR509119 | OR509064 |
| 15 | BP15 | *P. cf. lichen* | 5-P.cf.lichen |  | UOG Biorepository | IZ00044 | Guam, Pago Bay | OR509235 | OR509172 | OR509120 | OR509065 |
| 16 | BP16 | *P. lobata* | 7-P.compressa- P.murrayensis-like |  | UOG Biorepository | IZ00045 | Guam, Pago Bay | OR509236 | OR509173 | 0 | OR509066 |
| 17 | BP17 | *P. cf. evermanni* | 1-P.annae-P.evermanni-like |  | UOG Biorepository | IZ00046 | Guam, Pago Bay | OR509237 | OR509174 | OR509121 | OR509067 |
| 18 | BP18 | *P. lobata* | 6-P.lobata-like |  | UOG Biorepository | IZ00047 | Guam, Pago Bay | 0 | OR509175 | OR509122 | OR509068 |
| 19 | BP19 | *P. cf. murrayensis* | 6-P.lobata-like |  | UOG Biorepository | IZ00048 | Guam, Pago Bay | OR509238 | OR509176 | OR509123 | OR509069 |
| 20 | BP20 | *P. cf. evermanni* | 1-P.annae-P.evermanni-like |  | UOG Biorepository | IZ00049 | Guam, Pago Bay | OR509239 | OR509158 | OR509124 | OR509070 |
| 21 | FB04 | *P. rus* | 2-P.rus |  | UOG Biorepository | IZ00053 | Guam, Fouha Bay | OR509240 | OR509177 | 0 | OR509071 |
| 22 | FL01 | *P. lobata* | 9-P.australiensis-like |  | UOG Biorepository | IZ00090 | Guam, Fouha Bay | OR509241 | OR509178 | OR509125 | OR509072 |
| 23 | FL02 | *P. lobata* | 8-P.compressa- P.australiensis-like |  | UOG Biorepository | IZ00091 | Guam, Fouha Bay | OR509242 | OR509179 | 0 | 0 |
| 24 | FL06 | *P. lobata* | 9-P.australiensis-like |  | UOG Biorepository | IZ00095 | Guam, Fouha Bay | OR509243 | OR509180 | OR509126 | OR509073 |
| 25 | FL07 | *P. lobata* | 9-P.australiensis-like |  | UOG Biorepository | IZ00096 | Guam, Fouha Bay | OR509244 | OR509181 | OR509127 | OR509074 |
| 26 | FL08 | *P. lobata* | 7-P.compressa- P.murrayensis-like |  | UOG Biorepository | IZ00097 | Guam, Fouha Bay | OR509245 | OR509182 | OR509128 | OR509075 |
| 27 | FL09 | *P. lobata* | 9-P.australiensis-like |  | UOG Biorepository | IZ00098 | Guam, Fouha Bay | OR509246 | OR509183 | OR509129 | OR509076 |
| 28 | FL11 | *P. lobata* | 9-P.australiensis-like |  | UOG Biorepository | IZ100 | Guam, Fouha Bay | OR509247 | OR509184 | OR509130 | 0 |
| 29 | FL12 | *P. lobata* | 9-P.australiensis-like |  | UOG Biorepository | IZ101 | Guam, Fouha Bay | OR509248 | OR509185 | OR509131 | OR509077 |
| 30 | FL13 | *P. lobata* | 7-P.compressa- P.murrayensis-like |  | UOG Biorepository | IZ102 | Guam, Fouha Bay | OR509249 | OR509186 | OR509132 | OR509078 |
| 31 | FL15 | *P. lobata* | 9-P.australiensis-like |  | UOG Biorepository | IZ104 | Guam, Fouha Bay | OR509250 | OR509187 | OR509133 | OR509079 |
| 32 | FL27 | *P. lobata* | 9-P.australiensis-like |  | UOG Biorepository | IZ116 | Guam, Fouha Bay | 0 | OR509177 | OR509134 | 0 |
| 33 | FL38 | *P. lobata* | 7-P.compressa- P.murrayensis-like |  | UOG Biorepository | IZ127 | Guam, Fouha Bay | 0 | OR509178 | OR509135 | OR509080 |
| 34 | MZ01 | *P. annae* | 1-P.annae- P.evermanni-like |  | UOG Biorepository | IZ00001 | Guam, Merizo Pier | OR509251 | OR509188 | OR509136 | OR509081 |
| 35 | MZ02 | *P. cylindrica* | 10-P.cylindrica |  | UOG Biorepository | IZ00002 | Guam, Merizo Pier | OR509252 | OR509189 | OR509137 | OR509082 |
| 36 | MZ03 | *P. rus* | 2-P.rus |  | UOG Biorepository | IZ00003 | Guam, Merizo Pier | OR509253 | OR509190 | OR509138 | OR509083 |
| 37 | MZ04 | *P. cylindrica* | 10-P.cylindrica |  | UOG Biorepository | IZ00004 | Guam, Merizo Pier | OR509254 | OR509191 | OR509139 | OR509084 |
| 38 | MZ05 | *P. lobata* | 9-P.australiensis-like |  | UOG Biorepository | IZ00005 | Guam, Merizo Pier | OR509255 | OR509192 | OR509140 | 0 |
| 39 | MZ06 | *P. cf. lobata* | 7-P.compressa- P.murrayensis-like |  | UOG Biorepository | IZ00006 | Guam, Merizo Pier | OR509256 | OR509193 | OR509141 | 0 |
| 40 | MZ07 | *P. rus* | 2-P.rus |  | UOG Biorepository | IZ00007 | Guam, Merizo Pier | 0 | 0 | OR509142 | OR509085 |
| 41 | MZ08 | *P. cf. lutea* | 3-P.cf.lutea |  | UOG Biorepository | IZ00008 | Guam, Merizo Pier | OR509257 | OR509194 | OR509143 | OR509086 |
| 42 | MZ09 | *P. cylindrica* | 10-P.cylindrica |  | UOG Biorepository | IZ00009 | Guam, Merizo Pier | OR509258 | OR509195 | 0 | OR509087 |
| 43 | MZ10 | *P. cylindrica* | 10-P.cylindrica |  | UOG Biorepository | IZ00010 | Guam, Merizo Pier | OR509259 | OR509196 | 0 | 0 |
| 44 | MZ11 | *P. cylindrica* | 10-P.cylindrica |  | UOG Biorepository | IZ00011 | Guam, Merizo Pier | OR509260 | OR509197 | OR509144 | OR509088 |
| 45 | MZ12 | *P. lobata* | 7-P.compressa- P.murrayensis-like |  | UOG Biorepository | IZ00012 | Guam, Merizo Pier | OR509261 | OR509198 | 0 | 0 |
| 46 | MZ13 | *P. rus* | 2-P.rus |  | UOG Biorepository | IZ00013 | Guam, Merizo Pier | OR509262 | OR509199 | OR509145 | OR509089 |
| 47 | MZ14 | *P. lobata* | 8-P.compressa- P.australiensis-like |  | UOG Biorepository | IZ00014 | Guam, Merizo Pier | OR509263 | OR509200 | 0 | 0 |
| 48 | MZ15 | *P. annae* | 1-P.annae- P.evermanni-like |  | UOG Biorepository | IZ00015 | Guam, Merizo Pier | OR509264 | OR509201 | OR509146 | OR509090 |
| 49 | MZ16 | *P. lobata* | 8-P.compressa- P.australiensis-like |  | UOG Biorepository | IZ00016 | Guam, Merizo Pier | OR509265 | OR509202 | OR509147 | OR509091 |
| 50 | MZ18 | *P. rus* | 2-P.rus |  | UOG Biorepository | IZ00018 | Guam, Merizo Pier | OR509266 | OR509203 | OR509148 | OR509092 |
| 51 | MZ19 | *P. cylindrica* | 10-P.cylindrica |  | UOG Biorepository | IZ00019 | Guam, Merizo Pier | OR509267 | OR509204 | OR509149 | 0 |
| 52 | MZ20 | *P. annae* | 1-P.annae- P.evermanni-like |  | UOG Biorepository | IZ00020 | Guam, Merizo Pier | OR509268 | OR509205 | OR509150 | OR509093 |
| 53 | MZ21 | *P. rus* | 2-P.rus |  | UOG Biorepository | IZ00021 | Guam, Merizo Pier | OR509269 | OR509206 | OR509151 | OR509094 |
| 54 | MZ22 | *P. rus* | 2-P.rus |  | UOG Biorepository | IZ00022 | Guam, Merizo Pier | OR509270 | OR509207 | OR509152 | OR509095 |
| 55 | MZ23 | *P. rus* | 2-P.rus |  | UOG Biorepository | IZ00023 | Guam, Merizo Pier | OR509271 | OR509208 | OR509153 | OR509096 |
| 56 | MZ24 | *P. lobata* | 7-P.compressa- P.murrayensis-like |  | UOG Biorepository | IZ00024 | Guam, Merizo Pier | OR509272 | OR509209 | 0 | 0 |
| 57 | MZ25 | *P. rus* | 2-P.rus |  | UOG Biorepository | IZ00025 | Guam, Merizo Pier | OR509273 | OR509210 | OR509154 | OR509097 |
| 58 | MZ26 | *P. lobata* | 8-P.compressa- P.australiensis-like |  | UOG Biorepository | IZ00026 | Guam, Merizo Pier | OR509274 | OR509211 | 0 | OR509098 |
| 59 | MZ27 | *P. lobata* | 9-P.australiensis-like |  | UOG Biorepository | IZ00027 | Guam, Merizo Pier | OR509275 | OR509212 | 0 | OR509099 |
| 60 | MZ28 | *P. rus* | 2-P.rus |  | UOG Biorepository | IZ00028 | Guam, Merizo Pier | OR509276 | OR509213 | 0 | OR509100 |
| 61 | MZ30 | *P. cf. annae* | 1-P.annae- P.evermanni-like |  | UOG Biorepository | IZ00029 | Guam, Merizo Pier | OR509277 | OR509214 | 0 | OR509101 |
| 62 | OFL07 | *P. cf. evermanni* | 1-P.annae- P.evermanni-like |  | UOG Biorepository | IZ00056 | Guam, Fouha Bay | OR509278 | OR509215 | 0 | OR509102 |
| 63 | OFL08 | *P. cf. lutea* | 3-P.cf.lutea |  | UOG Biorepository | IZ00057 | Guam, Fouha Bay | OR509279 | OR509216 | 0 | OR509103 |
| 64 | OFL11 | *P. lobata* | 8-P.compressa- P.australiensis-like |  | UOG Biorepository | IZ00060 | Guam, Fouha Bay | OR509280 | OR509217 | OR509155 | OR509104 |
| 65 | OFL12 | *P. lobata* | 8-P.compressa- P.australiensis-like |  | UOG Biorepository | IZ00061 | Guam, Fouha Bay | OR509281 | OR509218 | OR509156 | OR509105 |
| 66 | OFL13 | *P. cf. lutea* | 3-P.cf.lutea |  | UOG Biorepository | IZ00062 | Guam, Fouha Bay | OR509282 | OR509219 | 0 | OR509106 |
| 67 | OFL15 | *P. lobata* | 9-P.australiensis-like |  | UOG Biorepository | IZ00064 | Guam, Fouha Bay | OR509283 | OR509220 | OR509157 | OR509107 |
|  |  |  |  |  |  |  |  |  |  |  |  |

| **Hawaii** | |  |  |  |  |  |  |  |  |  |  |
| --- | --- | --- | --- | --- | --- | --- | --- | --- | --- | --- | --- |
| 68 | Coral1_R | *P.lobata* | 6-P.lobata-like |  | Forsman et al. 2020 | SAMN06648852 | Hawaii, Oahu | X | X | X | X |
| 69 | Coral10_R | *P.lobata* | 7-P.compressa- P.murrayensis-like |  | Forsman et al. 2020 | SAMN06648859 | Hawaii, Oahu | X | X | X | X |
| 70 | Coral2_R | *P.lobata* | 7-P.compressa- P.murrayensis-like |  | Forsman et al. 2020 | SAMN06648853 | Hawaii, Oahu | X | X | X | X |
| 71 | Coral4_R | *P. evermanni* | 1-P.annae- P.evermanni-like |  | Forsman et al. 2020 | SAMN06648867 | Hawaii, Oahu | X | X | X | X |
| 72 | Coral5_R | *P. lobata* | 6-P.lobata-like |  | Forsman et al. 2020 | SAMN06648854 | Hawaii, Oahu | X | X | X | X |
| 73 | Coral6_R | *P. lobata* | 7-P.compressa- P.murrayensis-like |  | Forsman et al. 2020 | SAMN06648855 | Hawaii, Oahu | X | X | X | X |
| 74 | Coral7_R | *P. lobata* | 7-P.compressa- P.murrayensis-like |  | Forsman et al. 2020 | SAMN06648856 | Hawaii, Oahu | X | X | X | X |
| 75 | Coral8_R | *P. lobata* | 8-P.compressa- P.australiensis-like |  | Forsman et al. 2020 | SAMN06648857 | Hawaii, Oahu | X | X | X | X |
| 76 | Coral9_R | *P. lobata* | 7-P.compressa- P.murrayensis-like |  | Forsman et al. 2020 | SAMN06648858 | Hawaii, Oahu | X | X | X | X |
| 77 | L28pcommanual | *P. compressa* | 7-P.compressa- P.murrayensis-like |  | Forsman et al. 2020 | SAMN06648864 | Hawaii, Oahu | X | X | X | X |
| 78 | L62bl_R_001 | *P. evermanni* | 1-P.annae- P.evermanni-like |  | Forsman et al. 2020 | SAMN06648865 | Hawaii, Oahu | X | X | X | X |
| 79 | PCom1manual | *P. compressa* | 8-P.compressa- P.australiensis-like |  | Forsman et al. 2020 | SAMN06648861 | Hawaii, Oahu | X | X | X | X |
| 80 | PCom2 | *P. compressa* | 7-P.compressa- P.murrayensis-like |  | Forsman et al. 2020 | SAMN06648862 | Hawaii, Oahu | X | X | X | X |
| 81 | PCom3manual | *P. compressa* | 8-P.compressa- P.australiensis-like |  | Forsman et al. 2020 | SAMN06648863 | Hawaii, Oahu | X | X | X | X |
| 82 | PeveR2_R_001 | *P. evermanni* | 1-P.annae- P.evermanni-like |  | Forsman et al. 2020 | SAMN06648866 | Hawaii, Oahu | X | X | X | X |
| 83 | PLob02manual | *P. lobata* | 6-P.lobata-like |  | Forsman et al. 2020 | SAMN06648850 | Hawaii, Oahu | X | X | X | X |
| 84 | PLob1manual | *P. lobata* | 7-P.compressa- P.murrayensis-like |  | Forsman et al. 2020 | SAMN06648849 | Hawaii, Oahu | X | X | X | X |
| 85 | PLob3manual | *P. lobata* | 6-P.lobata-like |  | Forsman et al. 2020 | SAMN06648851 | Hawaii, Oahu | X | X | X | X |
| 86 | R10prus_R_001 | *P. rus* | 2-P.rus |  | Forsman et al. 2020 | SAMN06648868 | Hawaii, Kona | X | X | X | X |
| 87 | 103_S8.combined | *P. compressa* | 8-P.compressa- P.australiensis-like |  | Forsman et al. 2020 |  | Hawaii, Oahu | X | X | X | X |
| 88 | 104A_S9.combined | *P. compressa* | 7-P.compressa- P.murrayensis-like |  | Forsman et al. 2020 |  | Hawaii, Oahu | X | X | X | X |
| 89 | 104B_S10.combined | *P. compressa* | 7-P.compressa- P.murrayensis-like |  | Forsman et al. 2020 |  | Hawaii, Oahu | X | X | X | X |
| 90 | 105_S1.combined | *P. compressa* | 7-P.compressa- P.murrayensis-like |  | Forsman et al. 2020 |  | Hawaii, Oahu | X | X | X | X |
| 91 | 106_S2.combined | *P. compressa* | 8-P.compressa- P.australiensis-like |  | Forsman et al. 2020 |  | Hawaii, Oahu | X | X | X | X |
| 92 | 107a_S3.combined | *P. compressa* | 8-P.compressa- P.australiensis-like |  | Forsman et al. 2020 |  | Hawaii, Oahu | X | X | X | X |
| 93 | 107b_S4.combined | *P. compressa* | 8-P.compressa- P.australiensis-like |  | Forsman et al. 2020 |  | Hawaii, Oahu | X | X | X | X |
| 94 | 108_S5.combined | *P. compressa* | 7-P.compressa- P.murrayensis-like |  | Forsman et al. 2020 |  | Hawaii, Oahu | X | X | X | X |
| 95 | 113_S4.combined | *P. compressa* | 7-P.compressa- P.murrayensis-like |  | Forsman et al. 2020 |  | Hawaii, Oahu | X | X | X | X |
| 96 | 114_S5.combined | *P. compressa* | 7-P.compressa- P.murrayensis-like |  | Forsman et al. 2020 |  | Hawaii, Oahu | X | X | X | X |
| 97 | 115_S1.combined | *P. compressa* | 7-P.compressa- P.murrayensis-like |  | Forsman et al. 2020 |  | Hawaii, Oahu | X | X | X | X |
| 98 | 116A_S2.combined | *P. compressa* | 8-P.compressa- P.australiensis-like |  | Forsman et al. 2020 |  | Hawaii, Oahu | X | X | X | X |
| 99 | 116B_S3.combined | *P. compressa* | 8-P.compressa- P.australiensis-like |  | Forsman et al. 2020 |  | Hawaii, Oahu | X | X | X | X |
| 100 | 117_S6.combined | *P. compressa* | 7-P.compressa- P.murrayensis-like |  | Forsman et al. 2020 |  | Hawaii, Oahu | X | X | X | X |
| 101 | 118_S7.combined | *P. compressa* | 8-P.compressa- P.australiensis-like |  | Forsman et al. 2020 |  | Hawaii, Oahu | X | X | X | X |
| 102 | 133_S3.combined | *P. compressa* | 8-P.compressa- P.australiensis-like |  | Forsman et al. 2020 |  | Hawaii, Oahu | X | X | X | X |
| 103 | 134_S4.combined | *P. compressa* | 7-P.compressa- P.murrayensis-like |  | Forsman et al. 2020 |  | Hawaii, Oahu | X | X | X | X |
| 104 | 139_S9.combined | *P. compressa* | 8-P.compressa- P.australiensis-like |  | Forsman et al. 2020 |  | Hawaii, Oahu | X | X | X | X |
| 105 | 140_S10.combined | *P. compressa* | 8-P.compressa- P.australiensis-like |  | Forsman et al. 2020 |  | Hawaii, Oahu | X | X | X | X |
| 106 | 61_S6.combined | *P. compressa* | 8-P.compressa- P.australiensis-like |  | Forsman et al. 2020 |  | Hawaii, Oahu | X | X | X | X |
| 107 | 62_S7.combined | *P. compressa* | 7-P.compressa- P.murrayensis-like |  | Forsman et al. 2020 |  | Hawaii, Oahu | X | X | X | X |
| 108 | 63a_S3.combined | *P. compressa* | 8-P.compressa- P.australiensis-like |  | Forsman et al. 2020 |  | Hawaii, Oahu | X | X | X | X |
| 109 | 63b_S4.combined | *P. compressa* | 8-P.compressa- P.australiensis-like |  | Forsman et al. 2020 |  | Hawaii, Oahu | X | X | X | X |
| 110 | 64a_S5.combined | *P. compressa* | 8-P.compressa- P.australiensis-like |  | Forsman et al. 2020 |  | Hawaii, Oahu | X | X | X | X |
| 111 | 64b_S6.combined | *P. compressa* | 8-P.compressa- P.australiensis-like |  | Forsman et al. 2020 |  | Hawaii, Oahu | X | X | X | X |
| 112 | 67_S8.combined | *P. compressa* | 7-P.compressa- P.murrayensis-like |  | Forsman et al. 2020 |  | Hawaii, Oahu | X | X | X | X |
| 113 | 68_S9.combined | *P. compressa* | 7-P.compressa- P.murrayensis-like |  | Forsman et al. 2020 |  | Hawaii, Oahu | X | X | X | X |
| 114 | 73_S7.combined | *P. compressa* | 7-P.compressa- P.murrayensis-like |  | Forsman et al. 2020 |  | Hawaii, Oahu | X | X | X | X |
| 115 | 74_S8.combined | *P. compressa* | 8-P.compressa- P.australiensis-like |  | Forsman et al. 2020 |  | Hawaii, Oahu | X | X | X | X |
| 116 | 81_S8.combined | *P. compressa* | 7-P.compressa- P.murrayensis-like |  | Forsman et al. 2020 |  | Hawaii, Oahu | X | X | X | X |
| 117 | 85_S1.combined | *P. compressa* | 8-P.compressa- P.australiensis-like |  | Forsman et al. 2020 |  | Hawaii, Oahu | X | X | X | X |
| 118 | 93_S1.combined | *P. compressa* | 7-P.compressa- P.murrayensis-like |  | Forsman et al. 2020 |  | Hawaii, Oahu | X | X | X | X |
| 119 | 94_S2.combined | *P. compressa* | 7-P.compressa- P.murrayensis-like |  | Forsman et al. 2020 |  | Hawaii, Oahu | X | X | X | X |
| 120 | 95A_S5.combined | *P. compressa* | 8-P.compressa- P.australiensis-like |  | Forsman et al. 2020 |  | Hawaii, Oahu | X | X | X | X |
| 121 | 95B_S6.combined | *P. compressa* | 8-P.compressa- P.australiensis-like |  | Forsman et al. 2020 |  | Hawaii, Oahu | X | X | X | X |
| 122 | 96_S7.combined | *P. compressa* | 7-P.compressa- P.murrayensis-like |  | Forsman et al. 2020 |  | Hawaii, Oahu | X | X | X | X |
| 123 | *haborpor* | ***P. sp*.1* harbour** | 17-P. sp. harbour |  | Forsman et al. 2020 |  | Hawaii, Oahu | X | X | X | X |
| 124 | L24pbrigmanual | ***P.* sp.2*** | 6-P.lobata-like |  | Forsman et al. 2020 | SAMN06648860 | Hawaii, NWHI | X | X | X | X |
| 125 | L25psup_R_001 | ***P*. sp.3* encrusting** | 4-*P*. sp. encrusting |  | Forsman et al. 2020 | SAMN06648869 | Palmyra Atoll | X | X | X | X |
|  |  |  |  |  |  |  |  |  |  |  |  |
| **NCBI (n = 11)** | |  |  |  |  |  |  |  |  |  |  |
| 126 | NC037434 | *P. fontanesii* | *15-P.fontanesii* |  | NCBI | NC037434 | Yemen; Socotra Island | X | X | X | X |
| 127 | NC037435 | *P. harrisoni* | *12-P.harrisoni* |  | NCBI | NC037435 | Saudi Arabia | X | X | X | X |
| 128 | KU572435 | *P. lobata* | 7-P.compressa- P.murrayensis-like |  | NCBI | KU572435 | Hawaii | X | X | X | X |
| 129 | KU761954 | *P. lobata* | 6-P.lobata-like |  | NCBI | KU761954 | Costa Rica (TEP^1^) | X | X | X | X |
| 130 | KU159432 | *P. lutea* | 3-P.cf.lutea |  | NCBI | KU159432 | China: Hainan Island | X | X | X | X |
| 131 | JF825142 | *P. okinawensis* | 7-P.compressa- P.murrayensis-like |  | NCBI | JF825142 | *unknown* | X | X | X | X |
| 132 | KJ546638 | *P. panamensis* | 11-P.panamensis- P.sverdrupi |  | NCBI | KJ546638 | Mexico (TEP^1^) | X | X | X | X |
| 133 | KU761953 | *P. panamensis* | 11-P.panamensis- P.sverdrupi |  | NCBI | KU761953 | Costa Rica (TEP^1^) | X | X | X | X |
| 134 | DQ643837 | *P. porites* | 14-P.porites |  | NCBI | DQ643837 | USA: Florida | X | X | X | X |
| 135 | LN864762 | *P. rus* | 2-P.rus |  | NCBI | LN864762 | *unknown* | X | X | X | X |
| 136 | KU956960 | *P. sverdrupi* | 11-P.panamensis- P.sverdrupi |  | NCBI | KU956960 | Mexico: Baja California (TEP^1^) | X | X | X | X |
|  |  |  |  |  |  |  |  |  |  |  |  |
|  |  |  |  |  |  |  |  |  |  |  |  |
| **Outgroups (n = 7)** | |  |  |  |  |  |  |  |  |  |  |
| 137 |  | *Goniopora columna* | Outgroup |  | NCBI | JF825141 |  | X | X | X | X |
| 138 |  | *Dendrophyllia arbuscula* | Outgroup |  | NCBI | KR824937 |  | X | X | X | X |
| 139 |  | *Dendrophyllia cribrosa* | Outgroup |  | NCBI | JQ290080 |  | X | X | X | X |
| 140 |  | *Tubastraea coccinea* | Outgroup |  | NCBI | JQ290078 |  | X | X | X | X |
| 141 |  | *Tubastraea coccinea* | Outgroup |  | NCBI | KX024566 |  | X | X | X | X |
| 142 |  | *Tubastraea tagusensis* | Outgroup |  | NCBI | KX024567 |  | X | X | X | X |
| 143 |  | *Turbinaria peltata* | Outgroup |  | NCBI | KJ725201 |  | X | X | X | X |

* Porites sp. 1 and 2 are named as in Forsman et al 2020. ^1^TEP = Tropical Eastern Pacific
