# Supplementary material for: Barcoding and mitochondrial phylogenetics of Porites corals
Source: PLoS One. 2024 Feb 15;19(2):e0290505. doi: 10.1371/journal.pone.0290505 (PMC10868756; doi:10.1371/journal.pone.0290505)
Supplement: S2 Table — (DOCX) [file pone.0290505.s002.docx]

### S2 Table: Primers

| **Primer** | **MT09** | **MT12** | **MT16** | **MT20** |
| --- | --- | --- | --- | --- |
| **Forward** | TTCAAACAAGTCGGTAAAAA | TAGCGGTTAAAATTCCTCAA | TGGAATGCTTTTATTTATACTCTC | AGTGCGGGGTCTGTTATT |
| **Reverse** | ATTAAGAGCCCATTTAGCAG | AGTGAAAATGTGGCTCCTAA | CATCCATTTTTACACCCAAG | ACGTACCAATGTCTTTATGGT |
| **PCR Tm** | **58ºC** | **61.5ºC** | **56ºC** | **63ºC** |

All 8 primer pairs were originally published in:

Paz-García DA, Galván-Tirado C, Alvarado JJ, Cortes J, García-De-León FJ, Hellberg ME, Balart EF 2016 Variation in the whole mitogenome of reef-building *Porites* corals. *Conservation Genetics Resources* 8:123–127. doi:10.1007/s12686-016-0527-x
