# Supplementary figures and images for: Barcoding and mitochondrial phylogenetics of Porites corals
Source: PLoS One. 2024 Feb 15;19(2):e0290505. doi: 10.1371/journal.pone.0290505 (PMC10868756; doi:10.1371/journal.pone.0290505)

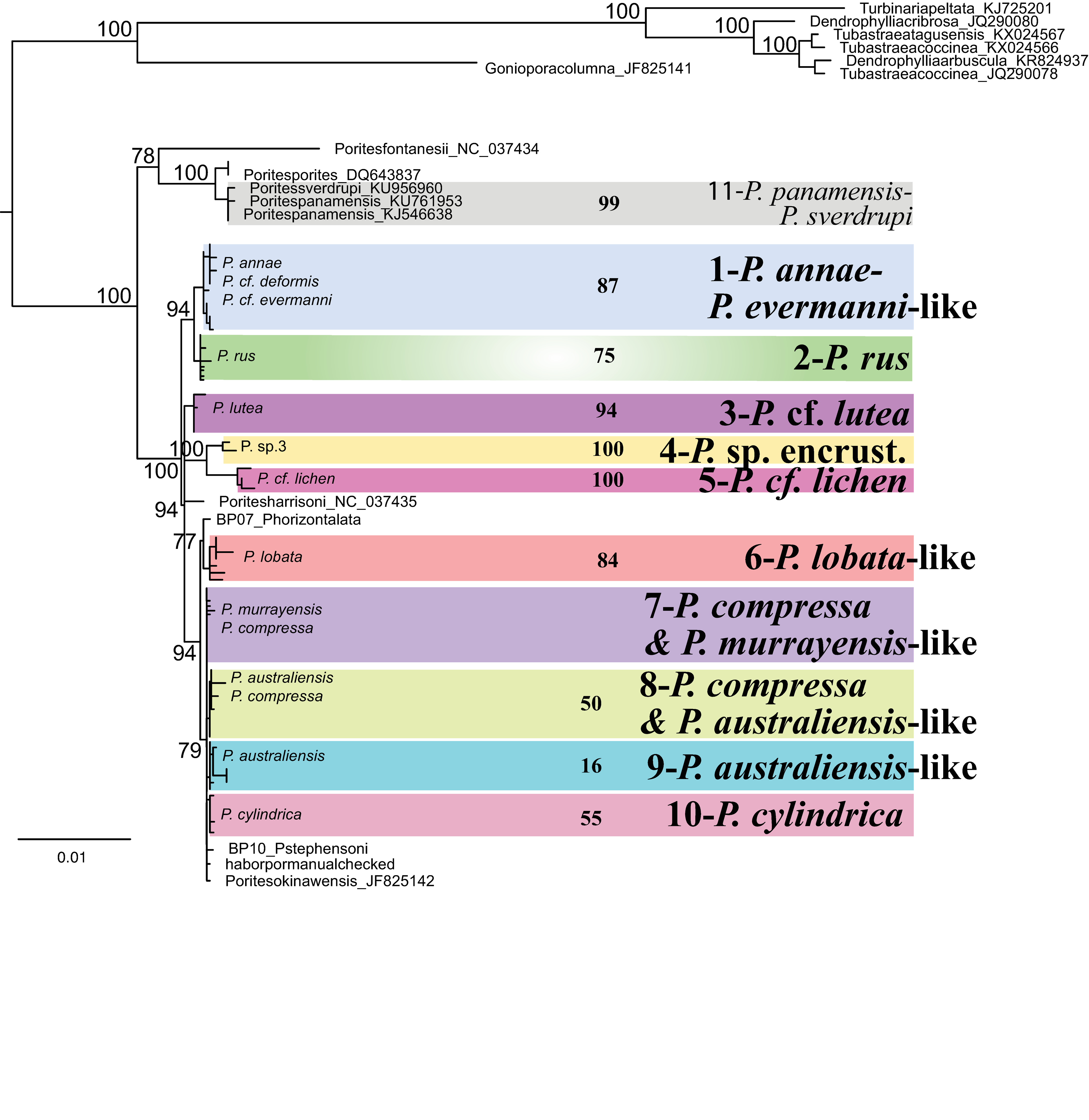

Supplement: S1 Fig — (TIF) [file pone.0290505.s003.tif]

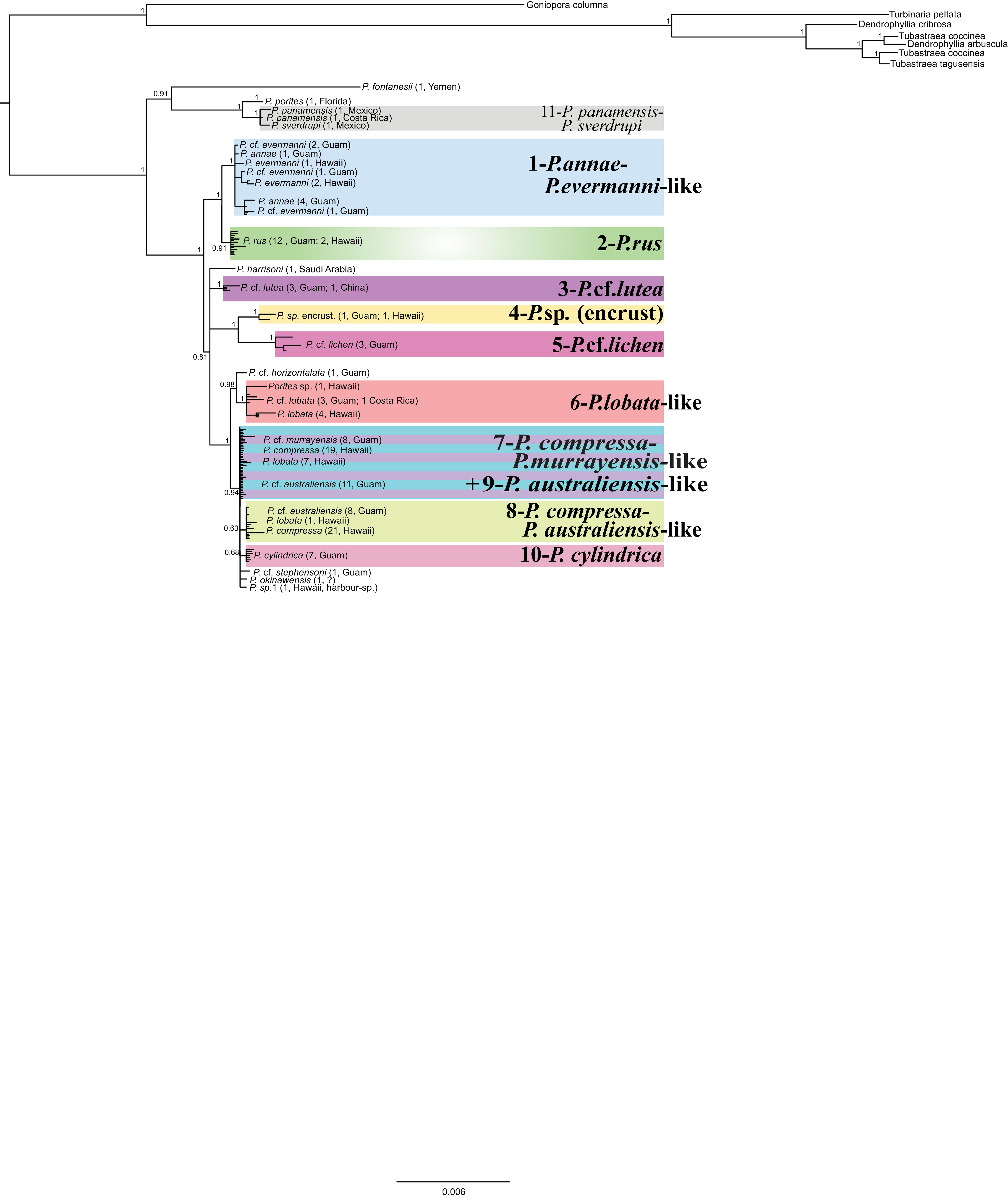

Supplement: S2 Fig — (TIF) [file pone.0290505.s004.tif]
